# Carers' attributions about positive events in psychosis relate to expressed emotion

**DOI:** 10.1016/j.brat.2009.06.004

**Published:** 2009-09

**Authors:** S.J. Grice, E. Kuipers, P. Bebbington, G. Dunn, D. Fowler, D. Freeman, P. Garety

**Affiliations:** a Department of Psychology, Institute of Psychiatry, Kings College London, P.O. Box 77, London SE5 8AF, UK; b Department of Mental Health Sciences, UCL, UK; c Health Methodology Research Group, School of Community Based Medicine, University of Manchester, UK; d School of Medicine, Health Policy and Practice, University of East Anglia, UK

**Keywords:** Attribution, Carers, Expressed emotion, LACS, Schizophrenia, Psychosis

## Abstract

**Background:**

Relapse is increased in people with psychosis who live with carers with high expressed emotion (EE). Attributional style has been used to understand EE at a psychological level. Previous studies have investigated carer appraisals for negative events in the patient's life. We therefore aimed to examine spontaneous carer attributions for both negative and positive events. Further, we distinguished between high EE based on critical comments, and that based on emotional-overinvolvement.

**Method:**

Audiotapes of the Camberwell Family Interview (CFI) (*N* = 70) were rated using the Leeds Attributional Coding System (LACS). Raters were blind to previous ratings of EE.

**Results:**

In our sample, low EE carers made significantly more attributions about positive events, and less about negative events than high EE carers. This is because criticism, but not overinvolvement, was strongly associated with responsibility attributions for negative events, while overinvolvement, but not criticism, was inversely associated with responsibility attributions for positive events.

**Conclusion:**

Carers' attributions for both positive and negative events may be a useful target for improving family interventions in psychosis.

## Introduction

### Expressed emotion

Expressed Emotion (EE) is a construct originally measured by rating how carers talk about the person they care for during a semi-structured interview, the Camberwell Family Interview (CFI) (Brown & Rutter, 1966; Vaughn & Leff, 1976). EE is of value because the risk of relapse of psychotic illness is greater in those whose carers display a high level of criticism, hostility or emotional-overinvolvement (EOI). These are termed high EE carers (Bebbington & Kuipers, 1994; Butzlaff & Hooley, 1998). Even during first episodes of psychosis, some carers perceive their situation as more stressful, use less helpful coping behaviours, and are rated as high EE (Raune, Kuipers, & Bebbington, 2004). Thus high EE is associated with increased distress not only in the patient but also in the carer.

### Attributions

An attributional model of EE based on differences in relatives' beliefs, including their ideas about intention and control, was first considered by Hooley (1985, 1987). As Hooley and Barrowclough discuss in their review (2003), this model also related to Greenley's observations about attributions of illness in carers of people with psychosis (Greenley, 1986). Brewin then went on to apply Weiner's more general theory (Weiner, 1985) of attribution, emotion and behaviour to an understanding of how EE might develop (Brewin, 1988; Brewin et al., 1991). Weiner had suggested that attributional appraisals of events were a way of understanding our emotional responses to them. We are likely to consider, firstly, whether an event is good or bad, and then to think about what might be the cause, why did it happen? Further, these attributions, Weiner has suggested, will determine how we then feel, and how we behave, and can be applied not only to events but to emotional responses to interactions within relationships. In the arena of mental health, for instance, attributional appraisals might be made about why someone with psychosis was being unresponsive. An attribution that they were being difficult, rather than that they were having a problem due to uncontrollable voices, would determine how a carer might understand the issues, how they might feel, and how they might react.

Brewin was the first to use the Leeds Attributional Coding System (LACS) to analyse previously recorded tapes of CFI interviews (Brewin, 1994; Brewin, MacCarthy, Duda, & Vaughn, 1991). The LACS is a systematic coding tool for analysing spontaneous causal attributions from transcripts of conversation (Munton, Silvester, Stratton, & Hanks, 1999; Stratton, Mutton, Hanks, Heard, & Davidson, 1986). Brewin et al. (1991) found that carers rated as high EE on the basis of criticism or hostility were more likely to make ‘controllable’ and ‘personal’ attributions than over-involved or low EE carers. The findings of a relationship between EE and relatives' attributions has since been corroborated, in carers of people with psychosis as well as in other disorders such as depression (Hooley & Licht, 1997; Wendel, Miklowitz, Richards, & George, 2000), dementia (Tarrier et al., 2002), diabetes (Wearden, Ward, Barrowclough, Tarrier, & Davies, 2006) and even conduct disorder in children (Bolton et al., 2003). They have also been replicated cross-culturally, including in China (Yang, Phillips, Licht, & Hooley, 2004).

The attributional studies suggest overall that high criticism carers (high CC) are more likely than low criticism (low CC) carers to believe that patients are substantially in control of the negative events that carers experience. Results from the LACS studies further suggest that carers rated as high EE because of critical comments more frequently ascribe to the patient, not only more control over negative events than low EE carers, but also more personal *responsibility* for them (Barrowclough & Hooley, 2003). This relationship is even greater when high EE is associated with a rating of hostility. Responsibility appraisals imply that events or behaviours are judged to be internal and personal to patients, *and* controllable by them. Thus, in relation to negative events, the most blaming causal attributions are linked to criticism and hostility (Barrowclough & Hooley, 2003).

However, carers who express high emotional-overinvolvement (EOI) have presented more difficulty for an attributional model of EE, such that some authors have simply dropped them from their analysis (Lopez et al., 2004), stating that ‘it appears that an attributional model of relapse does not apply to households characterised as high EOI’. Other authors have suggested that EOI may not be ‘attributionally mediated’ (Brewin et al., 1991). The problem of including EOI in analysis has been twofold: first, it is less common than criticism, and relatively fewer carers express EOI in the absence of criticism or hostility; second, studies that have used statistical or other methods to overcome this problem and include EOI in their analysis have found that these carers are similar to low EE carers (Barrowclough & Hooley, 2003).

Thus there is currently no reliable way of differentiating high EOI from low EE carers using an attributional model. However, there are indications in the literature to suggest that there is indeed an as yet unspecified difference in the attributional appraisals of these carers. For example, Barrowclough, Tarrier, and Johnston (1994) found a trend suggesting high EOI carers were even less likely than low EE carers to make responsibility (i.e. blaming) attributions for negative events. These carers were also noted to make more illness attributions i.e. citing the illness as the cause of the patient's difficulties more frequently, and thus blaming the patient and other factors less. Barrowclough et al. (1994) suggested that high EOI carers make sense of the patient's illness in terms of factors completely outside the patient's control. High EOI carers therefore attempt to change the course of events by their own behaviour, using themselves as a ‘buffer’ between the patient and the rest of the world. This is in keeping with previous suggestions in the literature that high EE should be described, not as a unitary construct, but as comprising two inherently different elements: criticism/hostility and EOI (Koenisberg & Handley, 1986).

The cultural context of EE may also be important, in that High EE (criticism) is extremely common in studies of Anglo-American and British families, but may be much less common in other cultures. Several studies have investigated EE, attributions and cultural context (Lopez et al., 2004; Weisman, Lopez, Karno, & Jenkins, 1993; Yang et al., 2004). Lopez et al. (2004) compared attributions of control and EE in Anglo-American and Mexican–American families. They found that two thirds of the former were classed as high EE, but only two fifths of the latter. For both ethnic groups, attributions of control were associated with EE, such that the belief that the patient could control their symptoms was associated with higher criticism and lower warmth. Interestingly, the study also found that warmth significantly reduced relapse in Mexican–American families, whereas in Anglo-American families criticism significantly increased it. This is of interest, since positive carer behaviours have rarely been predictive in Anglo-American/British studies. It is possible, however, that the study found protective effects of positive carer behaviours precisely because of the greater numbers of low EE families in the Mexican–American group, which increased statistical power. Lopez et al. (2004) suggested that more research was required to investigate low EE carers, and their attributes, coping styles and attributions.

The current study attempted to take all these issues into account. As suggested by Lopez et al. (2004), we wished to investigate in more detail the attributions of low EE carers of psychosis, using a relatively large sample. Given the high prevalence of warmth in low EE carers and our clinical experience, we thought it would be useful to examine attributions for positive as well as negative events. We also wanted to investigate ways of differentiating between low EE and high EOI carer responses, following the work of Barrowclough et al. (1994). Because the sample was relatively homogenous, the cultural issues discussed above would be unlikely to confound our findings.

We aimed, first, to investigate causal attributions made by carers about both *positive and negative* events in the patient's life. Secondly, we wished to find out if attributions about positive events would be made more frequently by low EE than by high EE carers. Finally, we wished to explore whether the pattern of attributions about positive and negative events, and patient responsibility for them, differed between low EE and high EOI carers. We were particularly interested in attributions rated as internal, personal, and controllable (henceforth called ‘responsibility attributions’).

On the basis of the evidence above, we hypothesized:

1 that carers would make attributions about positive as well as negative events during the course of the CFI;
2 that low EE carers would make more attributions about positive events than high EE carers;
3 that the balance of responsibility attributions in low EE carers would be shifted towards positive events compared with high EOI carers.

## Methods

The Psychological Prevention of Relapse in Psychosis (PRP) Trial (IS-RCTN83557988) was a UK multicentre randomised controlled trial of cognitive behavioural therapy and family intervention for psychosis designed to test hypotheses about outcome and about psychological processes associated with psychosis for both participants and carers (Garety et al., 2008).

The trial was located in four National Health Service (NHS) Trusts in London and East Anglia in the UK. Within each trust, recruitment was from specified inpatient and outpatient teams that agreed that all patients meeting the eligibility criteria would be asked to participate in the trial. These services were canvassed at least fortnightly for patients with psychosis who were relapsing. Patients meeting the inclusion criteria for the study (see below) were asked to provide informed consent. If they had carers, the one most in contact with the patient (i.e. for at least 10 hours a week, including telephone calls) was eligible for inclusion, provided they had been in a caring role for the patient for at least the previous 3 months, and had a command of the English language sufficient for interview and potential participation in psychological therapies. Consent was then sought from the patient for their carer to enter the trial. Carers were not approached unless this consent had been obtained. Only the primary carer was sought for each patient, and if consent was refused, no other carer from that family was approached.

Patients were recruited at the time of re-emergence of positive symptoms, either from a previously recovered state or from a state of persisting symptoms. For people with persisting symptoms, a significant exacerbation in positive symptoms was required, typically leading to hospital admission.

Patient inclusion criteria were:

Current clinical diagnosis of non-affective psychosis (schizophrenia, schizoaffective disorder, delusional disorder; IDC-10, F20); age 18–65 years, a second or subsequent episode starting not more than 3 months before a patient consented to enter the trial; a rating of at least four (moderate severity) on at least one positive psychotic symptom of the Positive and Negative Syndrome Scale (PANSS; Kay, 1991) at the first time of meeting.

The study design was cross sectional. The data were all obtained by trained independent research assessors, who interviewed patients and their carers, and administered questionnaires during the baseline phase of the randomized controlled trial and before allocation. Other baseline studies using data on carers and patients from this trial have already been published (Kuipers et al., 2006, 2007).

### Participants

In the PRP trial (Garety et al., 2008), 55% of patients did not consent to enter the treatment trial. No data thus exist on their caregivers. 94 patients with eligible caregivers did consent to enter the trial, but 11 of these caregivers did not. However, 3 of them gave consent for the initial assessments, including CFI interviews, which were completed at that stage. Thus 86/94 (92%) completed CFI interviews were available for this study. Of these, 70 (75%) provided audio tapes that could be used for the purposes of the current study; the remaining 16 were insufficiently audible for full transcription, were not complete, or had other technical problems Data analyses for this study are thus based on 70 carer CFI interviews.

### Measures

#### Camberwell Family Interview (CFI; Vaughn & Leff, 1976)

This is a semi-structured interview covering elements of family relationships and functioning, including arguments, symptoms and difficulties. Expressed Emotion (EE) is usually measured from an audio recording of the interview, which takes up to 2 h to administer (Brown & Rutter, 1966). Ratings involve extracting elements of what is said (i.e. content) as well as how it is said (i.e. prosody). Five scales are rated: Critical comments (frequency count); Emotional-Overinvolvement (EOI) (0–5); Hostility (score 0–3); Positive remarks (frequency count); Warmth (0–5). High criticism was defined as 6 critical comments or more, while high EOI was taken as a rating of 3 or above on the relevant scale. High EE was identified from high criticism, high overinvolvement, or the presence of any degree of hostility. The EE ratings used in this study had been previously completed, by trained raters blind to the current hypotheses. These raters were trained by Dr Christine Vaughn to rate EE reliably. High correlations or phi coefficients had been obtained by them on all EE scales: >0.76 for critical comments, EOI, hostility, warmth, positive remarks and overall EE category.

#### Attributions

Attributional statements were extracted from transcriptions of the 70 Camberwell Family Interviews using the LACS. This is a systematic coding tool that has been used extensively in EE research to code spontaneous attributions from the CFI (Munton et al., 1999; Stratton et al., 1986). The transcriber (SG) was blind to Expressed Emotion or any other ratings of the carers, and was not trained to rate Expressed Emotion. Carers identified specific positive or negative ‘events’ (classified as a behaviour, characteristic, or situation in the patient's life) and gave a causal explanation that reflected their own current opinion. Statements expressing the opinions of others, opinions not currently held, or hypothetical events were excluded. SG was trained in the use of the LACS following existing guidance (Stratton et al., 1986; Munton et al., 1999) and discussion with Professor Christine Barrowclough (personal communication), who also provided a manual.

The only difference in procedure was that positive as well as negative events were extracted (manual available from SG). For the purposes of the study, rateable positive events were those outcomes, behaviours or situations that were directly associated with the patient being discussed, and which the carer believed to be positive e.g. improvement of illness or reference to positive behaviours, feelings or characteristics of the patients, or current or past situations. The key criterion for coding whether an event was negative or positive was how the event was described by the carer, since the purpose of rating attributions is to assess the carer's appraisal of events. In this sense the rating is always contextual. Standard procedures from previous studies (Barrowclough & Hooley, 2003) were then adopted to calculate the reliability of extraction and rating of attributional statements. Seven transcribed CFI interviews (10% of the total) were randomly selected. SG then trained a second blind rater in all these procedures. SG and the second blind rater then extracted attributional statements independently from the CFI interviews, using the training materials and following the manual guidance about positive events written for the current study. A total of 195 attributional statements were extracted. 164 statements were common to both raters, giving a percentage agreement of 84%. Percentage agreement for individual transcripts ranged from 72% to 89%.

Following extraction, each statement was coded as to whether the event was negative or positive, and then in relation to four dimensions of patient causality. The *stable*/*unstable* dimension refers to whether the carer believes the cause is likely to persist and influence future events in the same way, or if it is likely to be transient or to fluctuate. The *internal*/*external* dimension refers to whether the carer believes the cause of the event is due to the operation of some psychological, behavioural or physical characteristic of the patient, or to some factor outside the patient (including most cases of illness, other people, or events in the outside world). The *personal*/*universal* dimension refers to whether the carer believes that something particular or idiosyncratic to the patient contributes to the outcome, or whether the it could be shared by typical members of the reference group (as defined by the carer). The final dimension, *controllable*/*uncontrollable*, refers to whether the carer believes the patient influenced the cause or could have changed the event without exceptional effort. Examples of attributions for positive and negative events and their dimensional coding are provided in Table 1.

For all causal dimensions, scores were dichotomized, and a score of 1 or 2 represented the poles of the dimension (1 stable, 2 unstable; 1 internal, 2 external; 1 universal, 2 personal; 1 controllable, 2 uncontrollable). A score of 9 was given if the attribution was uncodable because of insufficient information, or if the attribution held was a combination of both ends of the dimension. A further seven interviews were randomly selected when the extraction process was complete. From these, a total of 243 attributional statements were independently coded by the first author (SG) and the trained second rater. The inter-rater reliability of scoring on each of the dimensions was then calculated using the kappa statistic. These were as follows: Positive/Negative: *k* = 0.98; Stable/Unstable: *k* = 0.61; Internal/External: *k* = 0.89; Universal/Personal: *k* = 0.77; Controllable/Uncontrollable; *k* = 0.80. The inter-rater reliability statistics were interpreted with reference to Landis and Koch's (1977) criteria. The majority were classed as excellent, the remainder as acceptable.

For each carer, the number of responsibility attributions was calculated (i.e. the number of attributions coded internal, personal and controllable). A proportional attribution (PA) score for responsibility attributions, providing a score for each carer, was also calculated. The PA was derived by calculating the number of causes coded 1 on all of the dimensions (i.e. Internal, Personal and Controllable divided by the total number of causes. Causes coded 9 any of the dimensions were excluded from the analysis). PA scores thus range from 0 to 1, with scores closer to 1 indicating a greater proportion of individual scores coded as ‘responsible’. The PA was used instead of a simple frequency score, because we expected differences in the total number of attributions of each event type (positive/negative) across participant groups. This convention has been used in other studies for single attribution codings (Barrowclough et al., 1994).

#### Analysis

Analysis was carried out using SPSS for Windows (version 13). *T*-tests were used to compare means, while analysis of variance was used to assess the contribution of independent variables to the various attributions for events.

## Results

### Description of carer sample

This study was restricted to carers whose patients had consented that they were contacted, who had consented themselves, and then had provided interview tapes sufficiently complete and audible for total transcription using the available audio equipment. The demographic and clinical characteristics of the carer sample are presented in Table 2, and were very similar to that for the complete sample of carers (see Kuipers et al., 2006). Their mean age was 53 years (range 26–88). Most of the carers were female, with half being parents, and over 30% partners of patients. The majority classified themselves as White, were married, and were not employed outside the home. They had a mean total contact time with the patient of 79 hours/week (range 10–168), of which approximately 38 hours were face-to-face (range 7–84). Two thirds of carers were classed as low EE on the basis of the CFI. As in previous studies, only a small proportion of carers (7.1%) were classed as high EOI in the absence of criticism. However, after including those who also scored highly on criticism, there were enough carers with high EOI (17) to enable statistical analysis of the key hypotheses using analysis of variance (ANOVA). Only 3 carers were given a hostility rating, and hostility was never rated in the absence of criticism, so it was dropped from further analysis.

### Description of patient sample

The patient sample was also similar to that of Kuipers et al. (2006). They were predominantly male (*N* = 49 (70%)), with a long term history of psychosis including a recent relapse. The mean duration of illness was 11.2 years (range 1–44 years). Sixty (85.7%) were white, 59 (84.3%) unemployed and 53 (76.8%) unmarried. Sixty one (87%) had a diagnosis of schizophrenia, the remainder a diagnosis of schizoaffective disorder.

### Attributions

The 70 carers made a total of 2014 attributions. While the majority, 1437 (71.4%, mean = 20.5, S.D 8.8, range 6–46), were made for negative events, there were also 577 positive attributions (28.6%, mean = 8.2, S.D. 5.2, range 0–26). When high and low EE carers were compared (Table 3), there were no differences in the total numbers of attributions made. However, there was a clear difference in that low EE carers made significantly more attributions about positive events, and less about negative events than high EE carers.

Attributions for negative events were predominantly external (67.3%), unstable (68.9%), universal (68.5%) and uncontrollable (77.7%). Attributions for positive events were predominantly internal (60.5%), stable (61.4%), personal (60.5%) and controllable (52.2%).

**Fig. 1:**
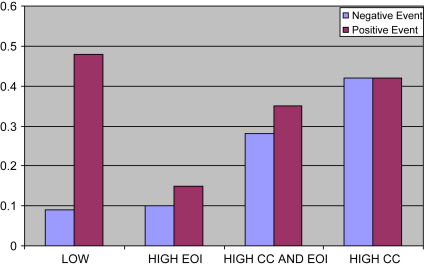
Mean carer responsibility (PA) scores by EE subgroups for positive and negative events.

The means displayed in Table 4 and Fig. 1 show the patterning of attributions about patient responsibility for both negative and positive events in the four groups defined by the presence or absence of EOI and of criticism. There is a noticeable difference between the four groups, including between the low EE group and the high EOI/low criticism group. Inspection suggests that EOI and criticism might have differential effects on attributions for positive and negative events.

To test this, criticism and EOI were entered into analysis of variance (ANOVA) as factors, with responsibility proportional attribution (PA) as the dependent variable. The first analysis was based on negative events, the second on positive events. There was a significant main effect of criticism on attributions for negative events indicating that high criticism carers characteristically make responsibility attributions for negative events (*F*(1,66) = 26.09, *p* < 0.0001). There was no main effect of EOI and no interaction. For positive events however, there was a main effect of EOI in reducing responsibility attributions (*F* (1,66) = 4.92, *p* = 0.03). There was no main effect of criticism on attributions for positive events, and no significant interaction.

To rule out the contribution of patient symptoms in this sample to carer attributions, we looked at these relationships. In Kuipers et al. (2006) there was a relationship between criticism in carers and patient anxiety, but not to symptoms of psychosis. In this sample there was no evidence that carer responsibility attributions related to patients' positive symptoms of psychosis, but some evidence of a relationship with patient affect. Carer responsibility attributions for positive events were negatively correlated with patient depression (*r* = −0.28, *p* = 0.02), while carer responsibility attributions for negative events were directly correlated to patient depression (*r* = 0.31, *p* = 0.01) and to patient anxiety (*r* = 0.46, *p* = 0.0001). As expected, carer criticism related to patient anxiety, and not to patient depression (anxiety, *r* = 0.32, *p* < 0.01; depression, *r* = 0.16, *p* = 0.2).

These results suggested that low EE carers make proportionately more responsibility attributions for positive than negative events in patients with symptoms of psychosis, compared to high EE carers. We tested this directly by calculating a difference score from the responsibility PA mean scores on positive and negative events. This difference was significantly greater for the low EE group (0.40) than for the high EE group (0.05, *t* (68) = 4.18, *p* = 0.0001).

## Discussion

This study investigated the relationship between attributions and EE in a relatively large sample of low EE carers of people with psychosis whose patients had consented to enter a treatment trial. In this sample we analysed attributions for positive as well as for negative events. The mean number of attributions for negative events (20) was in the mid-range of results from previous studies (range 17–23) (Barrowclough & Hooley, 2003). There was no difference between high and low EE carers in the total number of attributions. However, low EE carers made significantly more attributions about positive events (mean = 9.5 v 5.9), and fewer about negative events (mean = 18.7 v 24) than high EE carers. A previous study found that high EE (especially high EOI) carers make more attributions than low EE carers (Barrowclough et al., 1994). Our study suggests that this finding may have been due to the earlier focus on negative events in isolation.

Our other findings concerned responsibility attributions. These have previously been described as the most blaming attributions, as the cause is held to be ‘internal, personal, and controllable’. Our results lend support to our exploratory third hypothesis; high criticism carers made most responsibility attributions for events and high EOI carers fewest, even fewer than low EE carers. In part, this confirms Barrowclough et al.'s (1994) findings that high EOI carers made less responsibility attributions than low EE carers for negative events. However, because we included responsibility attributions for positive events, we were able to demonstrate that the responsibility attributions of low EE carers were predominantly in the positive domain.

It seems that high criticism is characterised by responsibility appraisals ascribed to both positive and negative events. High EOI on the other hand is associated with what might be called ‘victim appraisals’ of the patient, whereby carers rarely attribute responsibility to patients for anything. In contrast, Low EE carers are inclined towards ‘survivor appraisals’ of the patient, who is seen as less responsible for negative events, but more responsible for positive events. This may be protective for low EE carers. More ‘realistic’ high EE responses in carers are associated with high subjective burden, anxiety and depression (Scazufca & Kuipers, 1996). High impact of care was found to relate to feelings of stress and depression in carers in our recent study of mainly low EE carers (Kuipers et al., 2006).

These results suggest that the appraisals of low EE carers were more nuanced, more dependant on the event type than those of high EE carers. MacCarthy et al. (1986) found evidence of this tendency using a completely different methodology.

### Limitations

The EE ratings and attribution ratings were made from the same interview, introducing a danger of cross-contamination. However, evidence from other studies suggests the risk of this is unlikely to be great (Barrowclough et al., 1994; Wendel et al., 2000).

There were relatively few carers rated low on criticism and high on EOI, in this relatively sizeable sample. This limits the statistical power of our analyses. More seriously, a high proportion of our carers were low EE (64%), more than the 50% generally reported in the literature (Bebbington & Kuipers, 1994). It is not clear why our sample was different. It is likely to be a biased sample, because ethically we had to ask patients to consent to enter the trial before we could contact their carers. This requirement is not unusual in treatment trials of family interventions in psychosis (Szmukler et al., 2003), but may have led to our recruiting an unusual sample of carers. Measurement or rating error is unlikely to offer an explanation, due to rigorous training and the high inter-rater reliability of the EE ratings (Kuipers et al., 2006). Another possible explanation is that the sample of carers was largely drawn from non-inner-city locations such as Essex and Norfolk and this might explain the lower EE ratings. There may also have been a positive secular change in the nature of caring, perhaps due to the increased resources, awareness and support for carers of those with psychosis, compared to the earlier EE studies conducted in the 1980s. However, even though it is not clear why the sample was biased toward low EE ratings, it indicates a limitation on the generalisability of our results.

### Implications for clinical intervention

It is clear that caring for an individual with severe mental illness relates to high levels of distress and burden (Kuipers et al., 2006). As McFarlane and Cook (2007, p. 196) have recently pointed out, over time both patients and carers can find themselves in escalating negative cycles of interaction, fuelled by loss of hope and frustration, and a lack of effective coping and support. They emphasise that this is eminently treatable, and that ‘family members are as much victims of severe mental disorder as patients themselves’. However while family intervention for psychosis improves patient outcome (Pharoah et al., 2006; NICE guidelines' update for schizophrenia 2009), it has rarely included carer distress as its primary target. Given that patients and carers seem to form part of the same reflexive system, directing attention at carer distress as well as patient outcomes might be optimal. Some intervention studies have indeed tried to improve carer distress by taking carer needs into account (e.g. Szmukler et al., 2003), but have been disappointing, as carers have tended to show little change in burden or distress in relation to their caring role.

If we are to relieve carer distress through family or other interventions, we must consider what influences this distress. Some factors have already been identified e.g. coping style, social support, and carer appraisals of their experience. The current study identified a further factor: carers' attributions of patients' responsibility for positive events in their lives. It is possible that if carers were able to focus more on appraising patients as responsible for positive events in their lives, this might lead to improvements in carer expectations and patient functioning, less role stress and more positive interactions. Often implicit in family intervention, this could with advantage be made more explicit.

## Conclusion

In summary, the principal findings of the study were firstly, that carers do make spontaneous attributions for positive events; secondly, that low EE carers in our sample characteristically made more positive and fewer negative attributions than high EE carers; thirdly, that attributional responsibility appraisals for positive events were inversely related to EOI and those for negative events were associated specifically with criticism.

Thus we have shown that the behaviour of high EOI carers in this sample can be incorporated into an attributional framework that differentiates them from low EE carers; these low EE carers were more likely to attribute responsibility to patients for positive events. Trying to increase carer responsibility attributions for the positive events in patients' lives might form a useful part of future clinical intervention.

## Figures and Tables

**Table 1:** Examples of attributions for positive and negative events and their codings.

	Positive	Negative
Stable	He gets on with everyone at work because he is a really friendly person who can talk to anyone	He drinks as though it is going out of fashion because he is an excessive person
Unstable	He sleeps well but only when he is on the medication	He doesn't sleep when this psychosis is coming on
Internal	He got promoted after only six months because he was determined to succeed	He stopped the medication because he decided he didn't like the side effects
External	She is a lot better (more well) now because I looked after her at home and did everything for her	She hit me and threatened to kill herself; it was an episode, not my mum
Universal	He is very affectionate with me; he is a water sign and water signs are emotional and sensitive	His doesn't do anything round the house because he is lazy just like any other boy
Personal	He does loads round the house because he likes to help, he is the exception, some men just don't want to know	He is very underactive, and its not the medication, its him, he was always lazy
Controllable	He was enormous but he got himself really trim by watching what he eats	He lost a lot of money because he went out clubbing and took loads of drugs
Uncontrollable	He became really well and happy when they took him into hospital and gave him a depot	He went downhill dramatically because his father uprooted him away from his friends and took him to a new area

**Table 2:** Demographic and EE Variables for Carer Participants (*N* = 70).

Attribute	Frequency	Percentage
**Gender**		
Female	50/70	71.4
Male	20/70	28.6

**Ethnicity**		
White	62/69	89.9
Black/other	7/69	10.1

**Relationship to patient**		
Parent	37/70	52.9
Spouse	24/70	34.3
Other	9/70	12.9

**Employment status**		
Employed	27/67	40.3
Unemployed/other	40/67	59.7

**Marital status**		
Married	40/69	58.0
Single/other	29/69	42.0

**Expressed emotion**		
Low	46/70	65.7
High: EOI	5/70	7.1
High: CC/hostility	7/70	10.0
High: CC/hostility and EOI	12/70	17.1

**Table 3:** Number of attributions for high EE (*N* = 24) and low EE (*N* = 46) carers.

		Mean	SD	
Attributions about	Low EE	9.5	4.7	*t* = 2.82, *p* = 0.006
Positive events	High EE	5.9	5.4	

Attributions about	Low EE	18.7	4.7	*t* = 2.49, *p* = 0.015
Negative events	High EE	24	9.1	

Total attributions	Low EE	30	10.7	*t* = −0.64, *p* = 0.52
	High EE	28.2	12.1	

**Table 4:** Mean (SD) responsibility attribution (PA) scores by EE group for positive and negative events.

Responsibility attribution PA	Low EE *N* = 46	High EE *N* = 24		
		LO CC HI EOI *N* = 5	HI CC HI EOI *N* = 12	HI CC LO EOI *N* = 7
Negative event	0.09 (0.13)	0.10 (0.11)	0.28 (0.14)	0.42 (0.26)
Positive event	0.48 (0.27)	0.15 (0.17)	0.35 (0.26)	0.42 (0.32)
